# Acylcarnitine Profiling in Meningiomas with Different NF2 Mutation Statuses

**DOI:** 10.3390/ijms26041570

**Published:** 2025-02-13

**Authors:** Joanna Bogusiewicz, Jacek Furtak, Marcin Birski, Krystyna Soszyńska, Anna Majdańska, Agata Ryfa, Marek Harat, Barbara Bojko

**Affiliations:** 1 Department of Pharmacodynamics and Molecular Pharmacology, Faculty of Pharmacy, Collegium Medicum in Bydgoszcz, Nicolaus Copernicus University in Torun, 85-089 Bydgoszcz, Poland; 2 Medical Faculty, Bydgoszcz University of Science and Technology, 85-796 Bydgoszcz, Poland; 3 Department of Neurosurgery, 10th Military Research Hospital and Polyclinic, 85-681 Bydgoszcz, Poland; 4 Laboratory of Clinical Genetics and Molecular Pathology, Department of Medical Analytics, 10th Military Research Hospital and Polyclinic, 85-681 Bydgoszcz, Poland

**Keywords:** acylcarnitine, solid-phase microextraction, meningioma, merlin, NF2

## Abstract

The mutation in NF2 is the most common alteration associated with meningioma oncogenesis, and it is related to the loss of a suppressing protein called merlin. At the same time, alterations in energy production are visible in cancer cells, where increased demands for energy are observed. Fatty acid oxidation could be one of the ways cancer cells obtain energy. This metabolic pathway uses the acylcarnitine shuttle system, which is responsible for the acylation of fatty acids and their transport through the mitochondria. Therefore, this study aimed to profile acylcarnitines with short, medium, and long acyl chain lengths in meningiomas to assess their changes in tumors with different NF2 mutation statuses. For the analysis, solid-phase microextraction (SPME) coupled with liquid chromatography–high-resolution mass spectrometry (LC-HRMS) was used. The presented sampling method enabled less invasive and easy collection of the analytes from the studied lesions, which can be crucial for future analysis of potential biomarkers in the surgery room. It was observed that higher levels of these analytes characterized meningiomas with NF2 mutations. Moreover, the increased energy consumption and elevated levels of acylcarnitines show that these analytes can be considered markers of increased fatty acid oxidation in NF2 mutated cells.

## 1. Introduction

Meningiomas are the most common benign brain tumors. The treatment is based on surgery; however, the tumor either cannot be entirely removed or is inaccessible in some cases. Also, it should be mentioned that some percentage of meningiomas can evolve into grade 2 or 3 tumors [1,2,3]. In this case, treatment is much more complex, and alternative therapies like chemotherapy or radiotherapy must be applied [1,2,3]. Therefore, basic research in the direction that enables an understanding of the relationships between genetic mutations, their translation to molecular biology, and, subsequently, their impact on the mechanisms behind the sudden increase in malignancy of meningiomas or resistance to a particular therapy is of great importance [2].

Research has revealed that a mutation in NF2 is the most common alteration associated with meningioma oncogenesis [4]. The protein encoded by this gene is merlin, which regulates cell adhesion and signaling and impacts tumorigenesis suppression [5]. A lack of merlin correlates with a higher incidence of multiple meningiomas and schwannomas in the central nervous system and a higher risk of developing malignant lesions [3,5]. Stepanova et al. observed that cells with an NF mutation have significantly higher fatty acid synthesis, described by the higher activity of FASN, ACC 1 and 2, and other enzymes in this metabolic pathway [5]. The authors also suspected some fatty acid oxidation alteration could be observed in samples lacking merlin. The explanation of this observation could be related to the phenomena described by Melone et al. as a “futile cycle”, where two metabolic cycles going in opposite directions could be used by cancer cells. Fatty acid biosynthesis supplies appropriate fatty acid levels, while their oxidation in the mitochondria provides energy for proliferating cells [6].

Intermediates that play a crucial role in fatty acid oxidation in mitochondria are acylcarnitines, esters of carnitine, and fatty acids [6,7]. The carnitine shuttle system evolved due to the impermeability of the mitochondrial membranes to fatty acids with long acyl chains [6,8]. Specialized enzymes, such as carnitine palmitoyltransferase 1 (CPT1) and 2 (CPT2), carnitine–acylcarnitine translocase (CACT), and carnitine acetyltransferase (CrAT), catalyze the reactions, enabling the transport of acyl chains of fatty acids through the mitochondrial membranes to the matrix, resulting in energy production as well as acyl chains’ catabolism [6,9]. Alterations in energy production are visible in cancer cells, where an increased demand for energy is observed [6]. Moreover, lipid metabolism is upregulated if glucose availability decreases to sustain growth and survival in unfavorable conditions [6]. Thus, the carnitine system plays an essential role in cancer metabolic plasticity. Studies of the carnitine shuttle system are usually related to assessing crucial enzyme activity, but reports on the profiling of direct acylcarnitine intermediates were also proposed [8,10]. However, the bottleneck of this approach is the need to homogenize the studied tissue and the time-consuming analysis. Therefore, methods that can cope with these problems are needed. Solid-phase microextraction (SPME) was one of the methods applied for acylcarnitines analysis in brain tumors [8].

SPME is based on the interaction between the sorbent coated on the small-size support (e.g., fiber with a diameter of ca. 200 µm) and the analytes in the analyzed sample [11]. The probe is introduced into the tissue for a particular time, after which it is stored or proceeds to the next step: desorption of the analytes into organic solution. Subsequently, samples can be analyzed using chromatography coupled with a mass spectrometer or other analytical instrumentation.

Linking the information on the impaired suppressor activity of merlin in NF2 mutant meningiomas and the role of fatty acid oxidation in energy production in cancerous tumors, acylcarnitine profiling was performed to test in greater detail if the acylcarnitine profile changes are dependent on different NF2 mutation statuses. Moreover, SPME probes were applied as a sampling method due to their reported low invasiveness to the patient, low toxicity to the environment, and the simplicity of the sampling procedure.

## 2. Results and Discussion

A set of several acylcarnitines was extracted from brain tumors using SPME fibers, and the relative standard deviation (RSD) was below 30%. Among the detected analytes were the short-chain acylcarnitines (SCACs) AC C2:0, AC C3:0, AC C4:0 and AC C5:0; the medium-chain acylcarnitines (MCACs) AC C6:0, AC C8:0, AC C10:0, AC C10:1, and AC C12:0; and the long-chain acylcarnitines (LCACs) AC C14:0, AC C14:1, AC C16:0, AC C16:1, AC C18:0, and AC C18:1 (Table 1). A similar set of analytes was extracted in a study on acylcarnitine profiling in gliomas, where SPME probes were also used for sampling [8]. Analysis was conducted in intact tissue to check if metabolite changes were observed directly in the studied sample. Due to the heterogenic metabolic nature of tumors at different malignancy levels, only benign tumors were included in the study. The goal was to assess the acylcarnitine profile in tumors with different NF2 mutation statuses and uniform malignancy levels. Moreover, to focus on only the changes in acylcarnitine profile related to different NF2 mutation statuses, samples without the mutation in the most common oncogenic drivers (AKT1, PIK3CA, TRAF, and KLF4) [12,13] were included. These changes can be observed in grade 1 and 2 tumors without mutations in NF2. Thus, this approach allowed the selection of tissue-specific potential biomarkers of tumors with different NF2 statuses.

**Figure 1:**
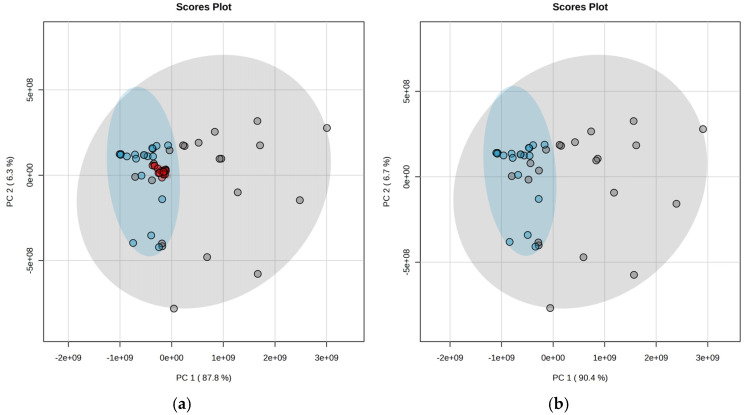
The PCA visualizes NFmt and NFwt meningiomas based on an acylcarnitine profile. (**a**) The PCA with pooled QC. (**b**) The PCA without pooled QC. QC—pooled quality control (red dots), NF2mt—NF2 mutated (gray dots), NF2wt—NF2 wildtype (cyan dots). Circles around studied groups display their 95% confidence regions.

A chemometric analysis using a principal component analysis (PCA) was conducted. Visualization of meningioma samples showed that NF2 mutant (NF2mt) tumors were more dispersed in the plot than NF2 wildtype (NF2wt) samples, which created a more concentrated group (Figure 1). The lack of merlin, a suppressor and microtubule stabilizer protein in NFmt meningiomas, could be related to more heterogenous metabolism and energy demands, impacting the acylcarnitine profile. The presence of an NF2 mutation can relate to a higher possibility of developing new foci of cancer origin or lead to tumor transformation or the occurrence of multiple tumors [14].

**Figure 2:**
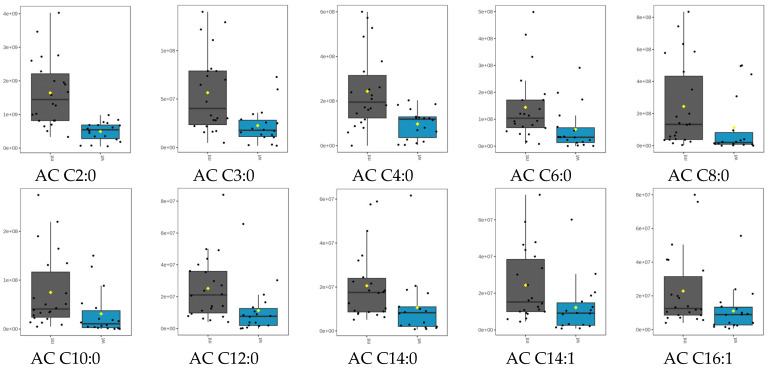
Box plots representing levels of significantly altered acylcarnitines in NF2 mutated and NF2 wildtype meningiomas analyzed using SPME coupled with LC-HRMS (*p* < 0.05). Plots for the raw data are given. NF2mt—NF2 mutated (gray boxes), NF2wt—NF2 wildtype (cyan boxes). The black dots represent the peak areas of the selected acylcarnitine from all samples. The notch shows the 95% confidence interval around the median of each group. The yellow diamond indicates the mean concentration of each group.

A trend of higher acylcarnitine levels in the samples lacking merlin (NF2mt) than in the wildtype was observed. This observation corresponds to the ratios of peak areas in NF2 mutant to NF2 wildtype meningiomas in Table 1, where levels of ten out of fifteen acylcarnitines were significantly different (*p* < 0.05) (Figure 2). Receiver operating characteristic (ROC) curves were prepared for the studied analytes, and it was observed that significantly changed acylcarnitines were characterized by AUCs higher than 0.7, confirming the potential of these analytes as biomarkers (Table 1, Figure S1). It should also be mentioned that the AUC for the model built on all studied analytes was 0.719, showing that acylcarnitine profiling can have diagnostic potential in differentiating tumors with different NF2 mutation statuses (Figure S2). Additionally, if drugs targeting lipid metabolism were applied, acylcarnitine profiling may have also been used as therapy assessment biomarkers. It should be pointed out that the possible activity of inhibitors of fatty acid synthetase (FASN) in NF2 mutant schwannoma cells was already postulated [15].

The lack of merlin can be related to higher malignancy and increased energy consumption, which can be observed in cancerous cells [16]. This demand could be fulfilled by changing glucose metabolism into aerobic glycolysis (the Warburg effect), elevated glutamine metabolism, or changes in fatty acid oxidation [5,6,17]. Indeed, it was reported in the literature that NF2 mutant cells are characterized by a higher dependence on lipid metabolism [5]. Thus, elevated acylcarnitine levels could explain the increased energy consumption and fatty acid oxidation in NF2 mutated cells. It should be mentioned herein that higher levels of acylcarnitines were observed in various types of cancerous lesions of glioma and breast cancer hepatocellular carcinoma in comparison with the respective non-cancerous samples [8,10,18,19,20].

Only benign tumors were studied in the studies presented herein. Further studies on extended cohorts containing higher-grade meningiomas are already planned. However, due to the rare incidence of these genetic changes [19], long-term studies to collect a representative study group are necessary. Also, it would be valuable to enrich the study by adding the appropriate genetic tests, such as CDKN2A homozygous deletion, used in third-grade meningioma diagnosis according to the WHO 2021 recommendations [21]. This is the marker of early recurrence and progression of the tumor, which is related to the inhibition of cyclin-dependent kinase 4 [22,23]. The exact genetic change was also observed in glioblastoma samples, which showed alterations in the lipid distribution and the content of oxidized fatty acids [24]. All these notes show the probable impact of CDKN2A homozygous deletion on lipid metabolism and, subsequently, fatty acid oxidation and acylcarnitine profile. Thus, acylcarnitine profiling with a consideration of CDKN2A status could be interesting. Another genetic alteration mentioned by the WHO 2021 recommendation is the change in the telomerase reverse transcriptase (TERT) gene promoter [21]. It was shown that the detected alteration in patients is related to higher neoplasm aggressiveness and lower patient survival [25]. Additionally, it should be noted that the analysis of post-translational modification, for instance, the loss of H3K27me3, can be impactful. This modification is associated with an increased risk of radiological recurrence for benign and low-malignancy WHO grade 1 and 2 meningiomas [26]. Possible alterations related to this modification can be related to the acylcarnitine profile due to its functions in mitochondrial metabolism, histone acetylation, and lipogenesis [27].

The results presented herein show changes in acylcarnitine levels in meningiomas with different NF2 mutation statuses. However, it would be beneficial to enrich this research with an assessment of the carnitine shuttle enzyme activity and the expression of genes responsible for their production. It would help to select the most important acylcarnitines in cancer diagnosis, especially considering the LCAC alteration, explained in the literature by the changes in CPT-2 activity [28,29,30,31]. Moreover, a method enabling fast and quantitative analysis should be introduced. The chromatographic analysis takes about half an hour to analyze one sample. Moreover, this time is even longer if the sample preparation is counted. Thus, to increase the chances of clinical use of acylcarnitine analysis in meningioma diagnosis, it would be useful to optimize the method to enable fast, quantitative, and reliable analysis of potential biomarkers. An additional advantage would be low invasiveness, as represented by the methods based on SPME. Therefore, technology such as coated-blade spray mass spectrometry (CBS) or microfluidic open interface (MOI) could be applied [32]. The CBS sampling is conducted with the probe, in the shape of a sword, coated with the sorbent at the tip. Then, the blade is mounted in the interface installed in the ion source. Subsequently, a drop of desorption solvent is added to the surface of the probe, and a high voltage is applied. Results could be acquired in a few seconds. CBS was tested for carnitine analysis in glioma homogenate as well as in the analysis of acylcarnitines in the intact meningioma tissue [33,34]. Another solution could be microfluidic open-ion-source MOI mass spectrometry based on the coated fiber sampling [35]. However, instead of desorption followed by instrumental analysis, the probe is put to the interface installed on the mass spectrometer. The interface consists of a chamber filled with desorption solvent. The probe is introduced into this chamber for a few seconds, during which desorption is conducted. Then, the solution with the desorbed analytes is directly injected into the mass spectrometer. The combination of desorption and instrumental analysis allows a reduction in analysis time.

As important as low invasiveness and the possibility of rapid analysis, introducing methods harmless to the environment can be crucial. Therefore, the objective factor, such as the ChlorTox, was calculated for the studied analytical platform. This parameter enables the estimation of substance toxicity in comparison with the standard substance—chloroform [25]. It was shown that the ChlorTox for solvents used for instrumental analysis is comparable with other methods presented in the literature [36]. Due to the wide application of homogenization followed by liquid–liquid extraction (LLE) in tissue analysis, the liquid–liquid extraction coupled with high-performance chromatography (LLE/HPLC) method given by Nowak et al. [32,36] was used as a reference. For instance, the ChlorTox for HPLC analysis was 3.36 g, while in the results presented herein, it was 3.87 g (Table 2) [36]. On the other hand, ChlorTox for the sample preparation protocol was significantly different. The ChlorTox was 2.78 g for the LLE-HPLC method compared with 0.21 g for the SPME method [36]. This observation shows that SPME as a sample preparation method is more environmentally friendly than LLE. It should be noted that ChlorTox per sample was calculated based on the number of studied samples, blanks, and QC samples. The data could also be biased due to limited information on the analytical methods for LLE/HPLC.

## 3. Materials and Methods

### 3.1. Chemicals and Materials

External calibrant Pierce LTQ Velos ESI Positive Ion Calibration Solution was purchased from Thermo Scientific. Isopropanol, methanol, water, acetonitrile, and ammonium acetate were LC-MS grade and were purchased from Merck (Warsaw, Poland). SPME C18 fibers were kindly provided by Supelco (Bellefonte, PA, USA).

### 3.2. Biological Material

Brain tumors were obtained during neurosurgical procedures in the 10th Military Research Hospital and Polyclinic in Bydgoszcz. SPME sampling was conducted directly after tumor removal. Meningothelial meningiomas without mutations in AKT1, PIK3CA, TRAF, and KLF4 were selected for this study: 22 tumors with a mutation in NF2 (NF2mt) and 18 samples without this genetic alteration (NF2wt). Only first-grade tumors were included in the analysis.

### 3.3. Genetic Testing

Tumor specimens were formalin-fixed and paraffin-embedded. All samples were classified by histopathological examination and graded according to WHO 2016 guidelines. DNA was extracted using the Maxwell 16 FFPE Plus LEV DNA Purification Kit and Maxwell 16 Instrument (Promega Corporation, Fitchburg, WI, USA). DNA samples were purified using the DNA Clean and Concentrator Kit (Zymo Research, Irvine, CA, USA). For multiplex ligation-dependent probe amplification (MLPA), DNA was isolated from the blood of healthy volunteers for use as controls.

MLPA and the SALSA MLPA P044-C1 kit (MRC-Holland, Amsterdam, the Netherlands) were used to detect loss (deletions) of the NF2 gene. MLPA assays were carried out by PCR according to the manufacturer’s protocol using 50 ng of normal and tumor DNA. Reference samples were included in each experiment. The PCR, DNA denaturation, and ligation steps were performed according to the manufacturer’s instructions. Amplified PCR products were separated by electrophoresis on an ABI PRISM 310 genetic analyzer (Thermo Fisher Scientific, Waltham, MA, USA), and, as an internal size standard, the LIZ-500 Genescan (Thermo Fisher Scientific) was used. Data were analyzed using the MRC-Coffalyser.Net (MRC-Holland).

Genotyping PCR reactions with TaqMan Universal PCR Master Mix (Thermo Fisher Scientific) and design (TaqMan SNP Genotyping Assays) or custom (Custom TaqMan Probes and Sequence Detection Primers) assays were used to detect mutations in the following genes: AKT1, PIK3CA (rs104886003, rs121913273, and rs121913279), TRAF (N520C, R653Q, R641C, and K615E), and KLF4. Genotyping assays were performed by PCR according to the manufacturer’s protocol using 10 ng of DNA template per reaction well on real-time thermal cycling instruments 7500RQPCR System (Thermo Fisher Scientific, Waltham, MA, USA).

### 3.4. Chemical Biopsy (Solid-Phase Microextraction) Protocol and LC-HRMS Analysis

Solid-phase microextraction probes coated with 7 mm C18 sorbent were used to sample brain tumors removed during the neurosurgical procedures. The fibers were preconditioned overnight in a methanol–water (1:1 *v*/*v*) solution, and then, directly before sampling, they were rinsed in water. Subsequently, the probe was inserted into the tissue for 30 min (extraction), and after this time, it was rinsed briefly in water. Probes were stored in a freezer at −30 °C until instrumental analysis. Then, the fibers were desorbed into 150 μL of isopropanol–methanol (1:1 *v*/*v*) solution using silanized inserts. Desorption was conducted for 1 h under agitation at 850 rpm [37]. Pooled quality control (QC) and extraction blanks were also prepared [37].

The liquid chromatography–high-resolution mass spectrometry (LC–HRMS) platform consisted of a Dionex UltiMate 3000 RS autosampler, a Dionex Ultimate 3000 RS pump (Thermo Fisher Scientific, Dionex, Bremen, Germany), and a Q Exactive Focus high-resolution mass spectrometer (Thermo Fisher Scientific, Bremen, Germany) was used for instrumental analysis.

LC analysis was conducted using 5 mM ammonium acetate in water as phase A and acetonitrile as phase B. Column: SeQuantZIC-cHILIC (3 μm 100 × 2.1 mm) was used, and the injection volume was set at 10 μL. The hydrophilic interaction chromatography (HILIC) was used in the analysis. The detailed parameters are given elsewhere [8]. The study was conducted in positive ion mode in a 100–1000 *m*/*z* scan range. Acylcarnitines were identified by matching their fragmentation patterns with spectra libraries at a mass accuracy of < 3 ppm (the presence of characteristic *m*/*z*: 85.0290 in MS/MS spectra). Full MS/dd-MS2 discovery mode was used for this purpose, and the detailed parameters of the fragmentation protocol are given elsewhere [8].

### 3.5. Data Processing and Statistical Analysis

Acylcarnitine identification was performed using XCalibur software 4.2.28.14 (Thermo Fisher Scientific, San Jose, CA, USA) based on *m*/*z* and characteristic fragmentation pattern. The peak areas for the obtained compounds were analyzed using MetaboAnalyst 6.0 and Statistica 13.3 PL software (StatSoft, Inc., Tulsa, OK, USA) [38]. Chemometric analysis, box-plot visualization, and receiver operating characteristic (ROC) curves were prepared. The average peak area, coefficient of variation, and the ratio of compared study groups for all analytes were calculated; the Mann–Whitney U test was applied to compare the variables. The *p*-value lower than 0.05 was set as the statistical significance threshold.

Finally, ChlorTox was calculated along with the recommendation given by Nowak et al. [36].

## 4. Conclusions

The application of SPME enabled simple profiling of a wide range of acylcarnitines in meningiomas and showed that the presence of NF2 mutation could alter the acylcarnitine profile. The loss of merlin coded by NF2 was related to a higher heterogeneity in the acylcarnitine profile and increased levels of detected carnitine esters. These results suggest that alterations in the acylcarnitine system could be crucial in assessing energy usage in cancerous cells and could serve as potential biomarkers of neoplastic changes in the diagnosis process or therapy response assessment. However, this observation has to be confirmed by tests conducted on a bigger group of patients. Moreover, applying SPME as a sampling and sample preparation method opens new possibilities for future applications and reduces environmental toxicity compared with the usually used methods, such as LLE.

## Figures and Tables

**Table 1:** The ratio of meningiomas with NF2 mutation to the tumor without this mutation was assessed using the SPME-LC-HRMS platform.

Acylcarnitine		*m*/*z*	RT	Raw Data			
				NF2mt/NF2wt Ratio	*p*-Value	FDR	AUC
SCAC	AC C2:0	204.1230	13.49	3.33	<0.05	<0.05	0.899
	AC C3:0	218.1387	11.94	2.50	<0.05	<0.05	0.785
	AC C4:0	232.1543	10.70	2.50	<0.05	<0.05	0.778
	AC C5:0	246.1700	9.92	2.28	0.206	0.219	0.619
MCAC	AC C6:0	260.1856	9.28	2.31	<0.05	<0.05	0.770
	AC C8:0	288.2169	8.60	2.16	<0.05	<0.05	0.760
	AC C10:0	316.2484	8.24	2.41	<0.05	<0.05	0.765
	AC C10:1	314.2326	8.29	1.77	0.066	0.066	0.672
	AC C12:0	344.2796	7.95	2.21	<0.05	<0.05	0.775
LCAC	AC C14:0	372.3108	7.75	1.94	<0.05	<0.05	0.742
	AC C14:1	370.2952	7.73	2.00	<0.05	<0.05	0.727
	AC C16:0	400.3423	7.63	1.63	0.055	0.074	0.679
	AC C16:1	398.3266	7.65	2.06	<0.05	<0.05	0.702
	AC C18:0	428.3734	7.63	1.11	0.219	0.219	0.616
	AC C18:1	426.3579	7.49	1.68	0.119	0.137	0.646

AC—acylcarnitine, AUC—area under the curve, FDR—false discovery rate, LCAC—long-chain acylcarnitine, MCAC—medium-chain acylcarnitine, NF2mt—NF2 mutated, NF2wt—NF2 wildtype, SCAC—short-chain acylcarnitines, RT—retention time.

**Table 2:** The calculation of the hazards of the SPME-LC-MS method in acylcarnitine analysis using HILIC chromatography and high-resolution mass spectrometry per sample.

Analysis Step	Reagents	CAS	CHsub	ChlorTox [g]	Total ChlorTox [g]
SPME	Methanol	67-56-1	4.81	0.16	0.21
	Isopropanol	67-63-0	3.13	0.05	
Instrumental Analysis	Ammonium acetate	631-61-8	0.00	0.00	3.87
	Acetonitrile	75-05-8	2.25	3.87	

## Data Availability

Spreadsheets with peak areas for acylcarnitines are presented in the Supplementary Materials. The raw files generated during the study presented herein are available from the corresponding author on reasonable request.

## Supplementary Materials

The following supporting information can be downloaded at: https://www.mdpi.com/article/10.3390/ijms26041570/s1.
