# Supplementary material for: Acylcarnitine Profiling in Meningiomas with Different NF2 Mutation Statuses
Source: Int J Mol Sci. 2025 Feb 13;26(4):1570. doi: 10.3390/ijms26041570 (PMC11855264; doi:10.3390/ijms26041570)
Supplement: Supplementary file 1 [file ijms-26-01570-s001.zip › Bogusiewicz_et_al_Suppl_file1.pdf]

Acylcarnitine profiling in meningiomas with different NF2 mutation status

Joanna Bogusiewicz [1], Jacek Furtak [2,3], Marcin Birski [3], Krystyna Soszyńska [4], Anna Majdańska [4], Agata Ryfa [4], Marek Harat [2,3]* and Barbara Bojko [1]*

[1] Department of Pharmacodynamics and Molecular Pharmacology, Faculty of Pharmacy, Collegium Medicum in Bydgoszcz, Nicolaus Copernicus University in Torun, Bydgoszcz, Poland
[2] Medical Faculty, Bydgoszcz University of Science and Technology, Bydgoszcz, Poland
[3] Department of Neurosurgery, 10th Military Research Hospital and Polyclinic, Bydgoszcz, Poland
[4] Laboratory of Clinical Genetics and Molecular Pathology, Department of Medical Analytics, 10th Military Research Hospital and Polyclinic, Bydgoszcz, Poland
* Correspondence: harat@10wsk.mil.pl (M.H.), bbojko@cm.umk.pl (B.B.).

SUPPLEMENTARY MATERIALS

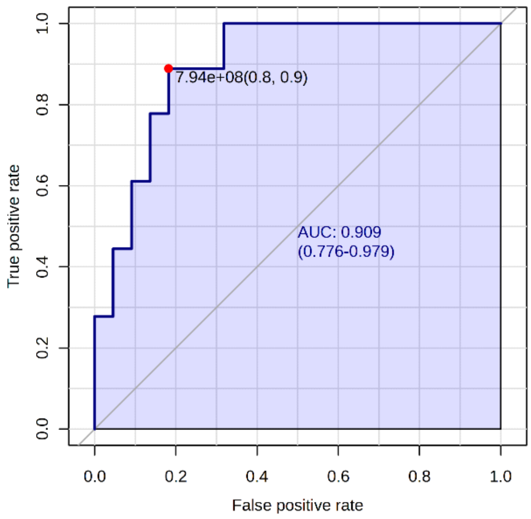

AC C2:0

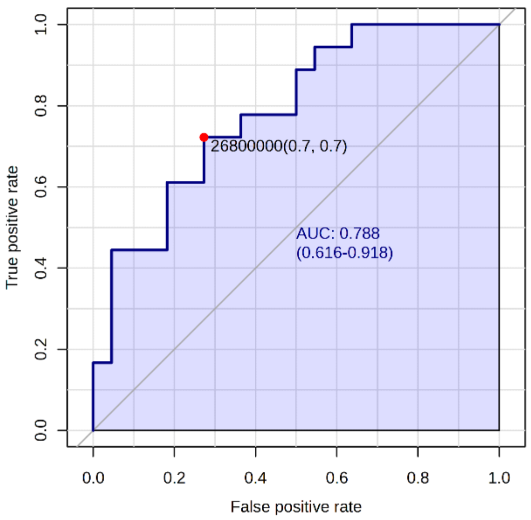

AC C3:0

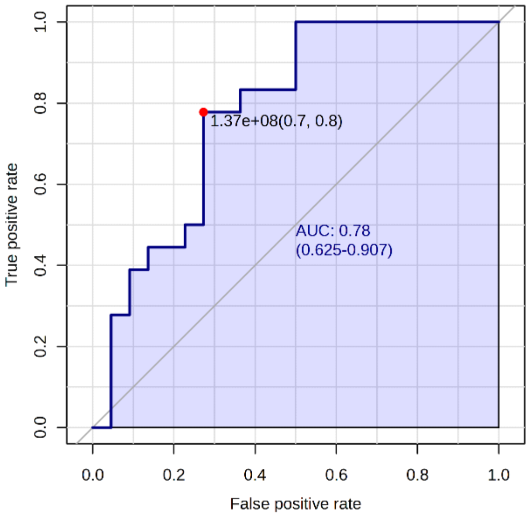

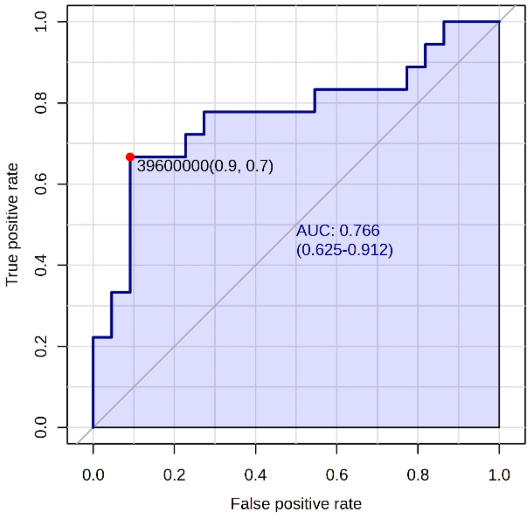

AC C4:0

AC C6:0

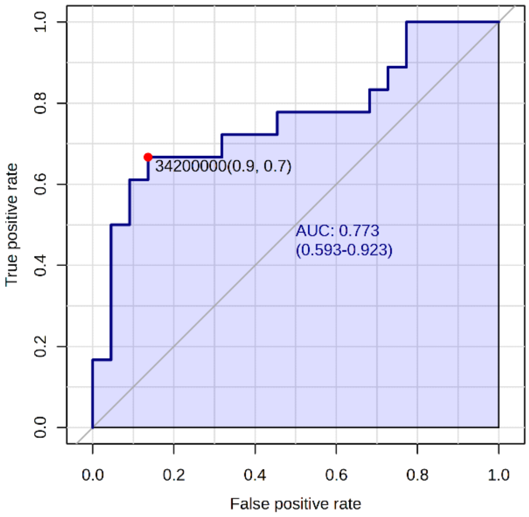

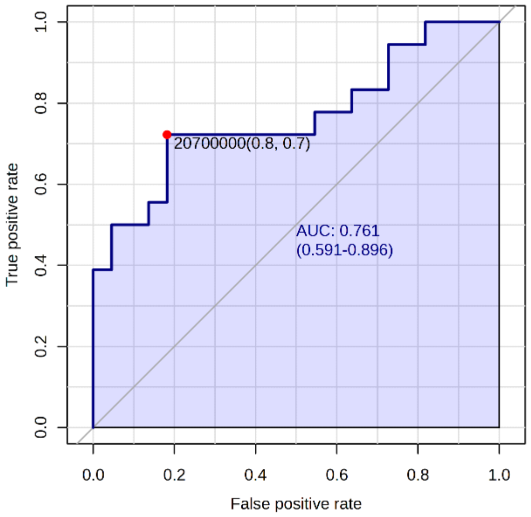

AC C8:0

AC C10:0

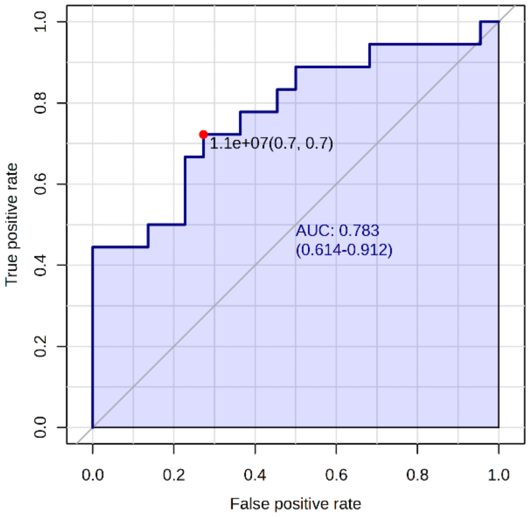

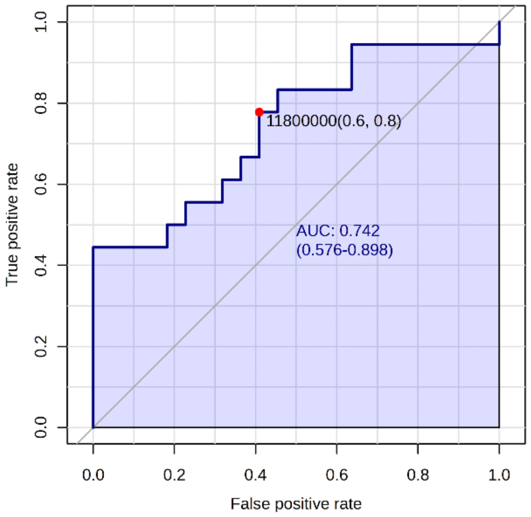

AC C12:0

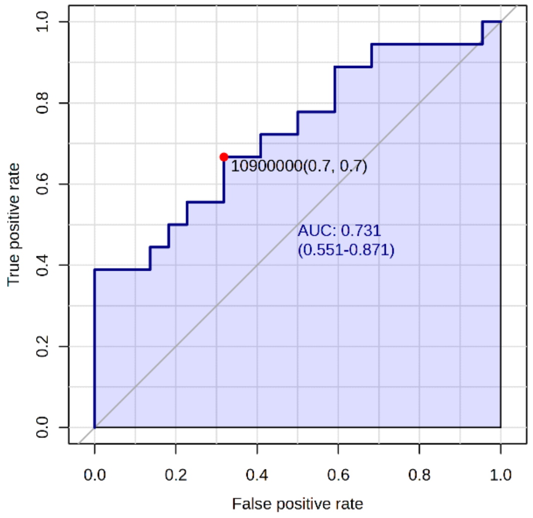

AC C14:0

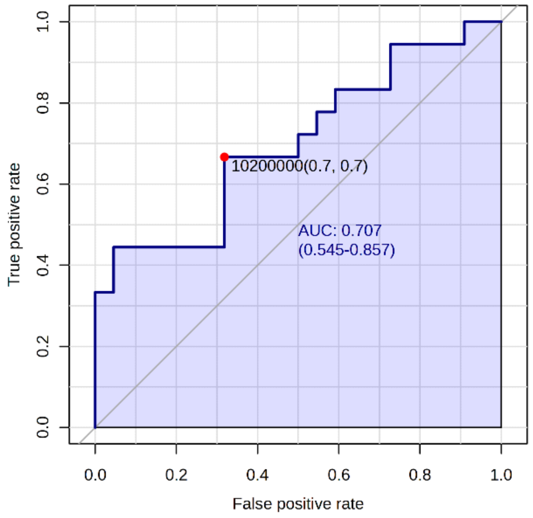

AC C14:1

AC C16:1

Figure S1. Receiver operating characteristic(ROC) curves for significantly altered acylcarnitines in NF2 mutated and NF2 wildtype meningiomas.

‘

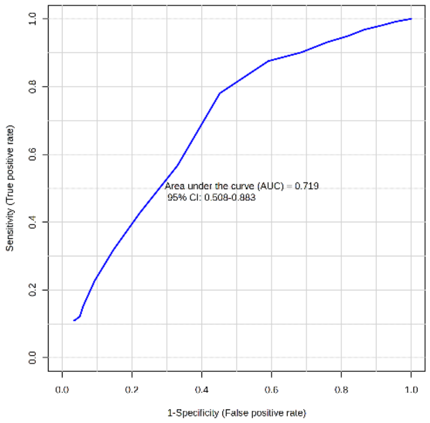

Figure S2. Receiver operating characteristic(ROC) curve for all studied acylcarnitines in NF2 mutated and NF2 wildtype meningiomas.
